# Analgesic effect of perineural magnesium sulphate for sciatic nerve block for diabetic toe amputation: A randomized trial

**DOI:** 10.1371/journal.pone.0176589

**Published:** 2017-05-02

**Authors:** Jiehao Sun, Xiaona Feng, Qihan Zhu, Wendong Lin, Hailei Guo, Emmanuel Ansong, Le Liu

**Affiliations:** 1 Department of Anesthesiology, 1^st^ Affiliated Hospital, Wenzhou Medical University, Wenzhou, China; 2 Department of Endocrinology, 1^st^ Affiliated Hospital, Wenzhou Medical University, Wenzhou, China; 3 Department of Burn, 1^st^ Affiliated Hospital, Wenzhou Medical University, Wenzhou, China; Mexican Social Security Institute, MEXICO

## Abstract

**Background and objectives:**

High concentrations of local anesthetics may be neurotoxic for diabetic patients. Additive perineural administration of magnesium was reported to decrease the consumption of local anesthetics for nerve block. It was hypothesized that MgSO_4_ added to dilute ropivacaine was equianalgesic to more concentrated ropivacaine for toe amputations in diabetic patients.

**Methods:**

Seventy diabetic patients were allocated into 3 groups: 1) perineural 200 mg MgSO_4_ added to 0.25% ropivacaine, 2) 0.25% ropivacaine alone, and 3) 0.375% ropivacaine alone. All patients underwent popliteal sciatic nerve block that was guided by ultrasonography using the respective regimens. Time of onset, duration of motor and sensory block were recorded. Spontaneous and evoked pain score, worst pain score, additional analgesic consumption, satisfaction score and initial time of analgesic requirement of each patient were documented up to 48 hours postoperatively.

**Results:**

In comparison with 0.25% ropivacaine alone, magnesium supplement prolonged the duration of sensory block (p = 0.001), as well as better evoked pain score at 6 hour postoperatively (p = 0.001). In comparison with evoked pain score (1.6/10) in group of 0.375% ropivacaine, magnesium plus 0.25% ropivacaine presented a little higher score (2.5/10) at 6 hour postoperatively (p = 0.001), while lower worst pain score (p = 0.001) and less postoperative total analgesic consumption (p = 0.002).

**Conclusions:**

The regimen of adding 200mg MgSO_4_ to 0.25% ropivacaine for sciatic nerve block yields equal analgesic effect in comparison with 0.375% ropivacaine. These findings have suggested that supplemental MgSO_4_ could not improve analgesic quality except reducing the total amount of local anesthetics requirement in diabetic toe amputations with sciatic nerve blocks.

## Introduction

Foot ulcer is one of the deleterious complications for diabetic patients. It is characterized by hyperalgesia [1] and can develops in 18.8% of patients [2]. Both angiopathy and neuropathy contribute to the development of diabetic foot ulcers. Hypomagnesemia was found to be associated with development of diabetic foot ulcer [3] and peripheral neuropathy [4].

The magnesium ion, a non-competitive N-methyl-D-aspartate (NMDA) antagonist, could improve postoperative analgesia in ultrasound-guided paravertebral block for patients undergoing thoracic surgery [5]. The mixture of local anesthetic and magnesium sulphate (MgSO_4_) for nerve block provided excellent tolerability and patient satisfaction, ensuring better postoperative analgesic effect and shield patients from the opioid related side effects [6]. Even oral magnesium supplementation could alleviate hyperalgesia in diabetic rats [7]. Gunduzet found that MgSO_4_ could provide dose-response analgesic effect in nerve block [8]. The dose of MgSO_4_ used in this study was based on the data from the two previous published studies [6,8]. We hypothesized that supplement of MgSO_4_ to perineural administration of ropivacaine for sciatic nerve block would a) decrease post-operative pain as well as severity of allodynia after toe amputation in diabetic patients who present with hyperalgesia and b) decrease the total amount of ropivacaine requirement to produce equivalent analgesic effect. The aim of this study was to investigate whether MgSO_4_ supplement of 0.25% ropivacaine could have equal analgesic effect in comparison with 0.375% ropivacaine on the sciatic nerve block for patient with diabetic foot ulcer undergoing toe amputation.

## Materials and methods

This prospective randomized, double blinded trial registered in Chinese Clinical Trial Registry (ChiCTR-TRC-14004944) was approved by the Ethics Committee of 1^st^ affiliated hospital of Wenzhou Medical University (Ethical number: 2014–34) on 26^th^ June 2014. Trial protocol as approved by the ethics committee was uploaded as S1 and S2 Files. Informed written consent was obtained from all patients before the study. Diabetic foot patients who had hyperalgesia to stimulus indicating toe amputation were recruited into the clinical trial. Doc Sun, Zhu, and Guo enrolled the participants. We gently probed the claimed toes to evaluate the evoked hyperalgesia. Examination to evoked hyperalgesia was made by means of a cotton swab and a Von Frey hair (no. 13, Somedic), respectively. We performed the stimulation on the skin overlying the center of 3^rd^ metatarsus. The patients were excluded if they had no evoked pain after stimulation. Other exclusion criteria included skin overlying the 3^rd^ metatarsus was not intact; patient incapacitation to the trial; systemic disease that contraindicated participation in the study; sedative abuse; opioid abuse or alcohol abuse; allergic to test drugs; age ≥70yr or ≤ 50 yr; unwilling to participate in the trial. The full trial protocol is registered at http://www.chictr.org.cn/showproj.aspx?proj=4629.

Upon hospitalization, patients were instructed by the anesthesiologist on the use of Numeric Rating Scale of Pain intensity–Visually (NRS) for assessing pain (0 cm = no pain, 10 cm = worst pain ever). On arrival at the operating room, intravenous access were established and routine monitoring of electrocardiogram, pulse oximetry, non-invasive blood pressure was applied. Supplemental oxygen was given by facemask (100% O2, 4 L/min) when required to maintain saturation above 92% throughout the duration of the study.

Each subject had an envelope with a code (generated from Microsoft Excel 2003 by doctor Sun) in it that was opened once the patient was enrolled. The envelops with codes were concealed at the Clinical Trial Center. A nurse anesthetist, who was not involved in patient assessment, looked at the codes and prepared the study regimen. Group R25 received 15ml of 0.25% ropivacaine; Group MR received the combination of 200mg MgSO_4_ and 0.25% ropivacaine with 15ml volume; Group R375 received 15ml of 0.375% ropivacaine. The following drugs were used: MgSO_4_ (10ml/2.5g. Adjuvant: normal saline. Batch number: 14503096, Shanghai Pharm Co. China), Ropivacaine (75mg/10ml Adjuvant: normal saline. Batch number: LANW,LAPK,LASB, AstraZeneca AB. UK). By using an ultrasound scanner with a 38 mm 6–13 MHz linear probe (EDGE, Fujifilm, SonoSite), 15 ml respective regimen was injected for each popliteal sciatic nerve block. Sciatic nerve was blocked just after bifurcation into common peroneal and tibial nerves. Both of two nerves identified within the sciatic nerve sheath were blocked guided by ultrasound with in-plane technique. Final needle located outside the perineural sheath in order to minimal nerve injury. Prior to surgery, no premedication was administered. An experienced anesthesiologist who performed the nerve block was unaware of which regimen was administered. Patients and nursing staffs were blinded to the group randomization.

Sensory block was assessed for sciatic nerve cutaneous innervation area by pinprick test in 3 categories (0: normal sensation; 1: no pain but presence of tactile sense; and 2: absence of any sensation). Motor block was assessed according to ankle dorsiflexion using a three point scale: 0 = normal dorsiflexion strength; 1 = diminished dorsiflexion strength; 2 = totally absent strength. The onset times of the sensory and motor blockades were defined as the time interval between the end of local anesthetic administration and the loss of sensation to pinprick (sensory score = 1) and absent movement (motor score = 2), respectively.

After surgery, an anesthetic nurse, who was blinded to patient allocation, evaluated the patients every 30 minutes until the time of complete regression of block. The time when patients were able to perform ankle dorsiflexion, was assumed as the end of motor block.

Hypotension was defined as systolic blood pressure < 90 mm Hg or > 30% decrease from baseline. Bradycardia was defined as < 50 beats/min. Intraoperative pain with NRS > 3 would be treated with increment dose of intravenous (i.v.) fentanyl 50ug.

Spontaneous pain intensity (NRS_rest_) and evoked pain intensity (assessed by stinging designated skin area with a cotton swab) (NRS_evoked_) were recorded by a trained anesthetist at 0, 6, 24, 48 hour after the operation. When evaluating the NRS_evoked_ score, the anesthetist applied the uniform pressure to the skin overlying the center of 3^rd^ metatarsus which was innervated by sciatic nerve (about 2 cm to the incision). It was the same area where we evaluated preoperative evoked hyperalgesia. The same anesthetist evaluated the NRS score throughout the trial to standardize the applied pressure in the trial.

The time from the completion of the surgery to the first request of tramadol was defined as patient’s initial time of analgesic requirement. Postoperatively, intravenous tramadol 50 mg or more was given for rescue analgesia whenever the NRS_rest_ > 3 or NRS_evoked_ >4. NRS value in worst pain state was documented according to the complaint from patients. Patients were asked to score their satisfaction on a scale from 0–100 two days (48 hours) after operation. Episodes of shivering and nausea were recorded during the study period. Shivering was assessed in 3 categories (0: no shivering; 1: no visible muscle activity but piloerection, peripheral vasoconstriction; 2: muscle activity in only one muscle group; 3: moderate muscular activity in more than one muscle group but no generalized shaking; 4: violent muscular activity that involves the whole body.). Nausea was assessed in 4 categories (0: no nausea; 1: slight nausea without vomit; 2: moderate nausea with vomit for 1–3 times; and 3: severe nausea with vomit for more than 3 times). Shivering and nausea were defined as the visible muscle activity (shivering score = 2) and slight nausea (nausea score = 1), respectively.

Data are shown as mean± SD or rate (%) of patients. Normal distribution of data was checked by the Kolmogorov–Smirnov test. Patient characteristics were compared using ANOVA test or Χ^2^ test, as appropriate. NRS scores were analyzed using the ANOVA test (in case of normal distribution) or Kruskal-Wallis test (in case of non-normal distribution).We calculated the AUC- NRS_rest_ (AUC-rest) and AUC- NRS_evoked_ (AUC-evoked) of NRS scores during 48h after operation according to the trapezium method by multiplying the time interval with the NRS score [9]. The patient’s initial time of analgesic requirement was estimated for each group with the use of Kaplan-Meier survival probabilities and compared with the log-rank test. Categorical data were assessed by the Χ^2^ test or Fisher’s exact test as appropriate. Multiple testing was performed using the Mann–Whitney U test or the Pearson Chi-square test, as appropriate. Results were considered statistically significant when the P-value was less than 0.05. In order to reduce type I error, p < 0.017 was considered statistically significant after Bonferroni correction in all possible multiple comparisons.

The primary outcome was the AUC-evoked (0-24h) value of NRS_evoked_ after the operation. Secondary outcomes included duration of block, time to first request of rescue analgesics, patient satisfaction and the worst pain score. We calculated that a sample size of 21 patients per group would achieve 80% power to detect a 20% reduction in AUC-evoked (0-24h) using one-way ANOVA [9]. Assuming a 10% drop rate, we recruited 25 patients to each group. Sample size estimates were done using PASS software (PASS 2008, Kaysville, UT, USA). Statistical analyses were done using SPSS 15.0 software (SPSS Inc., Chicago, IL, USA).

## Results

**Fig 1:**
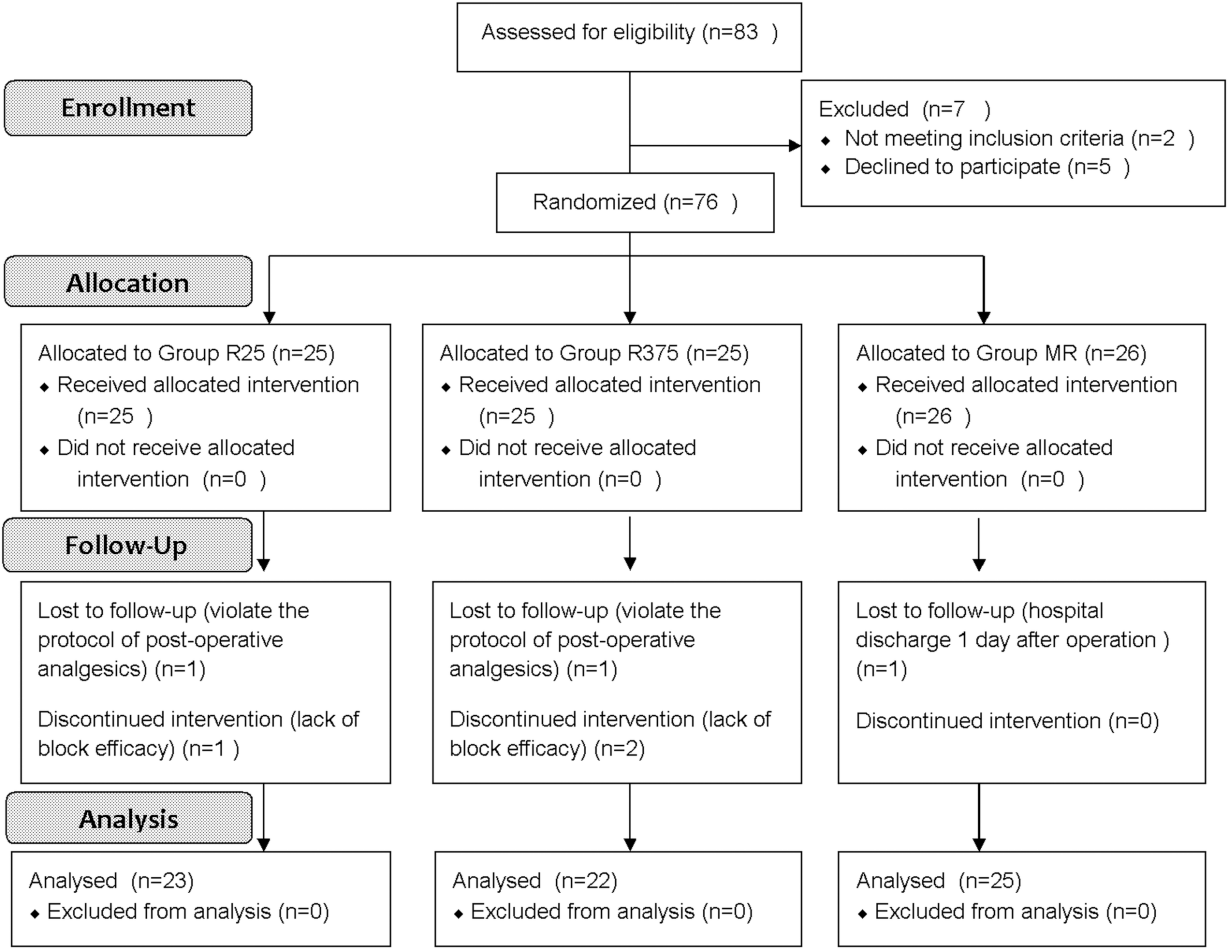
Flow of participants through the study.

A total of 70 patients were enrolled in the study, from June 2014 to October 2015 (Fig 1 for CONSORT flow diagram). The clinical trial was performed according to the CONSORT checklist (S1 Table). The recruitment was ended when we selected enough cases based on the sample size calculation. Demographical data was described in Table 1. BMI value in group R375 was less than the value in group MR (p = 0.03). Except for BMI, there were no significant differences between any of the groups for any of the demographic data.

**Table 1:** Characteristics of patients.

	R25 (n = 23)	MR (n = 25)	R375 (n = 22)	P value
Age (years)	60.5±5.0	59.7±5.3	62.2±5.0	0.243
BMI(kg/m^2^)	22.29±1.1	22.80±1.2	22.04±1.25	0.083
Male / Female	11 / 12	12 / 13	13 / 9	0.686
DM (years)	8.30±2.9	9.12±3.3	7.55±3.2	0.208
DM foot ulcer duration (day)	24[15,42]	27[15,51.5]	30.5[16,43.5]	0.375
Number of claimed toes	2[1,3]	2[1,3]	2[1,3]	1.000
Duration of surgery (min)	37.04±6.1	35.6±7.7	35.14±7.8	0.788
Serum Mg concentration (mmol/l)	0.79±0.06	0.78±0.07	0.77±0.06	0.697

BMI: Body mass index;

Values were expressed as number, mean ± SD or median [interquartile ranges].

R25: Perineural 0.25% ropivacaine with 15ml volume; MR: Perineural 200mg MgSO4 plus 0.25% ropivacaine with 15ml volume; R375: Perineural 0.375% ropivacaine with 15ml volume.

Same as Group R375, Group MR prolonged the duration of sensory block in comparison with Group R25 (p = 0.001). In comparison with other two groups, shorter time of onset and longer duration of motor block were detected in Group R375 (p = 0.001) (Table 2).

**Table 2:** Onset times, duration of sensory and motor blocks.

	R25 (n = 23)	MR (n = 25)	R375 (n = 22)	P value
Onset time of motor block (min)	10.7±1.8	10.8±1.8	8.6±1.1 *	0.001
Onset time of sensory block (min)	8.5±1.7	8.4±1.7	6.4±1.0 *	0.001
Duration of motor block (min)	298.7±29.9	312.8±26.5	374.5±26.0 *	0.001
Duration of sensory block (min)	524.3±47.7	612.4±42.6 †	640.9±43.9 †	0.001

Values were expressed as mean ± SD.

R25: Perineural 0.25% ropivacaine with 15ml volume; MR: Perineural 200mg MgSO4 plus 0.25% ropivacaine with 15ml volume; R375: Perineural 0.375% ropivacaine with 15ml volume.

* P<0.01 indicates a significant difference in comparison to Group R25 and Group MR.

† P<0.01 indicates a significant difference in comparison to Group R25.

**Fig 2:**
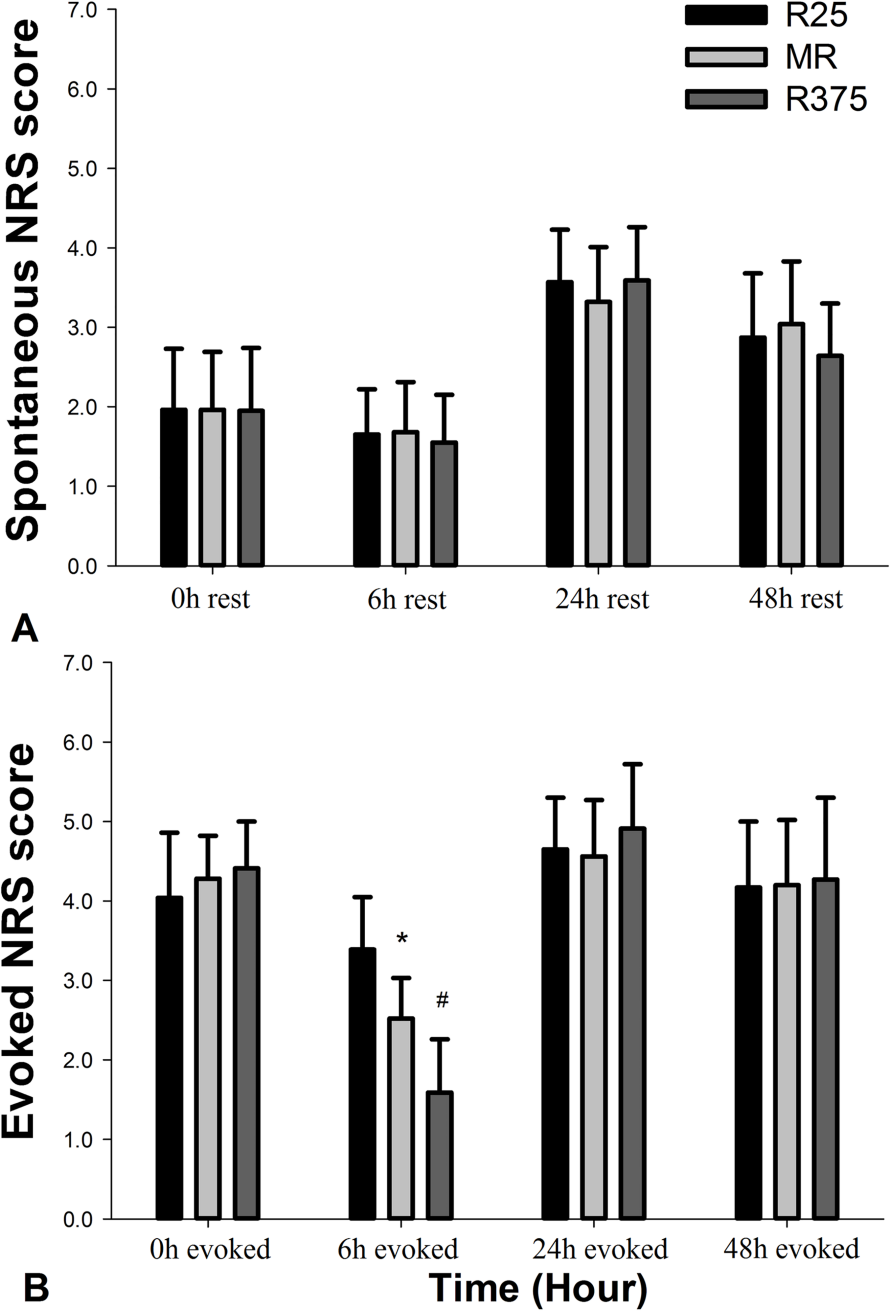
Pain scores (NRS) of the patients at rest and allodynia. (A) spontaneous NRS score; (B) evoked NRS score. Groups: R25: Perineural 0.25% ropivacaine with 15ml volume; MR: Perineural 200mg MgSO4 plus 0.25% ropivacaine with 15ml volume; R375: Perineural 0.375% ropivacaine with 15ml volume. Pain scores are presented as Mean ± SD. * P<0.01 indicates a significant difference in comparison to Group R25. # P<0.01 indicates a significant difference in comparison to Group R25 and Group MR.

Postoperative NRS pain scores were summarized in Fig 2. There were no significant differences among the three groups in NRS_rest_ after operation. For the evoked pain scores, there were also no significant differences in NRS_evoked_ among the three groups except at 6^th^ hour after operation. Group MR decreased the value of NRS_evoked_ at 6^th^ hour after the operation in comparison with Group R25 (p = 0.001). Group R375 provided lower NRS_evoked_ value at 6^th^ hour postoperatively in comparison with the other two groups (p = 0.001). (Fig 2).

The postoperative area under the curve (AUC_-evoked_ 0-24h) for the NRS_evoked_ values were lower in Group MR and Group R375 than that of Group R25 (p = 0.004 and p = 0.001, respectively). There was no statistical difference between the Group MR and Group R375 in AUC-evoked 0-24h (p = 0.056). (Table 3).

**Table 3:** Characteristics of analgesia and side effect.

	R25 (n = 23)	MR (n = 25)	R375 (n = 22)	P value
Number requiring rescue analgesic: N	21/23	20/25	19/22	0.532
Consumption of tramadol(0-24h)	50[50,100]	50[0,50] *	50[50,50] *	0.000
Consumption of tramadol(0-48h)	100[50,100]	50[50,75] *	100[50,100]	0.000
AUC-rest (0-24h) cm.h	57.7±9.1	55.4±9.9	57.5±8.2	0.000
AUC-rest (0-48h) cm.h	133.6±16.4	129.5±18.1	132.1±17.6	0.001
AUC-evoked (0-24h) cm.h	96.6±12.6	85.2±9.0 *	77.7±10.8 *	0.000
AUC-evoked (0-48h) cm.h	206.7±25.4	192.4±21.5	191.2±24.7	0.000
NRS in worst pain state	5.30±0.6	4.80±0.5†	5.32±0.6	0.000
Satisfaction score	68.83±3.4	86.44±2.9*	82.81±2.7*	0.000
Side effect: N				
Shivering	1/23	0/25	0/22	0.355
Nausea	4/23	5/25	3/22	0.846

Data were expressed as mean ± SD, median [interquartile ranges], or number of the patients (N).

R25: Perineural 0.25% ropivacaine with 15ml volume; MR: Perineural 200mg MgSO4 plus 0.25% ropivacaine with 15ml volume; R375: Perineural 0.375% ropivacaine with 15ml volume.

* P<0.01 indicates a significant difference in comparison to Group R25.

† P<0.05 indicates a significant difference in comparison to Group R25 and Group R375.

**Fig 3:**
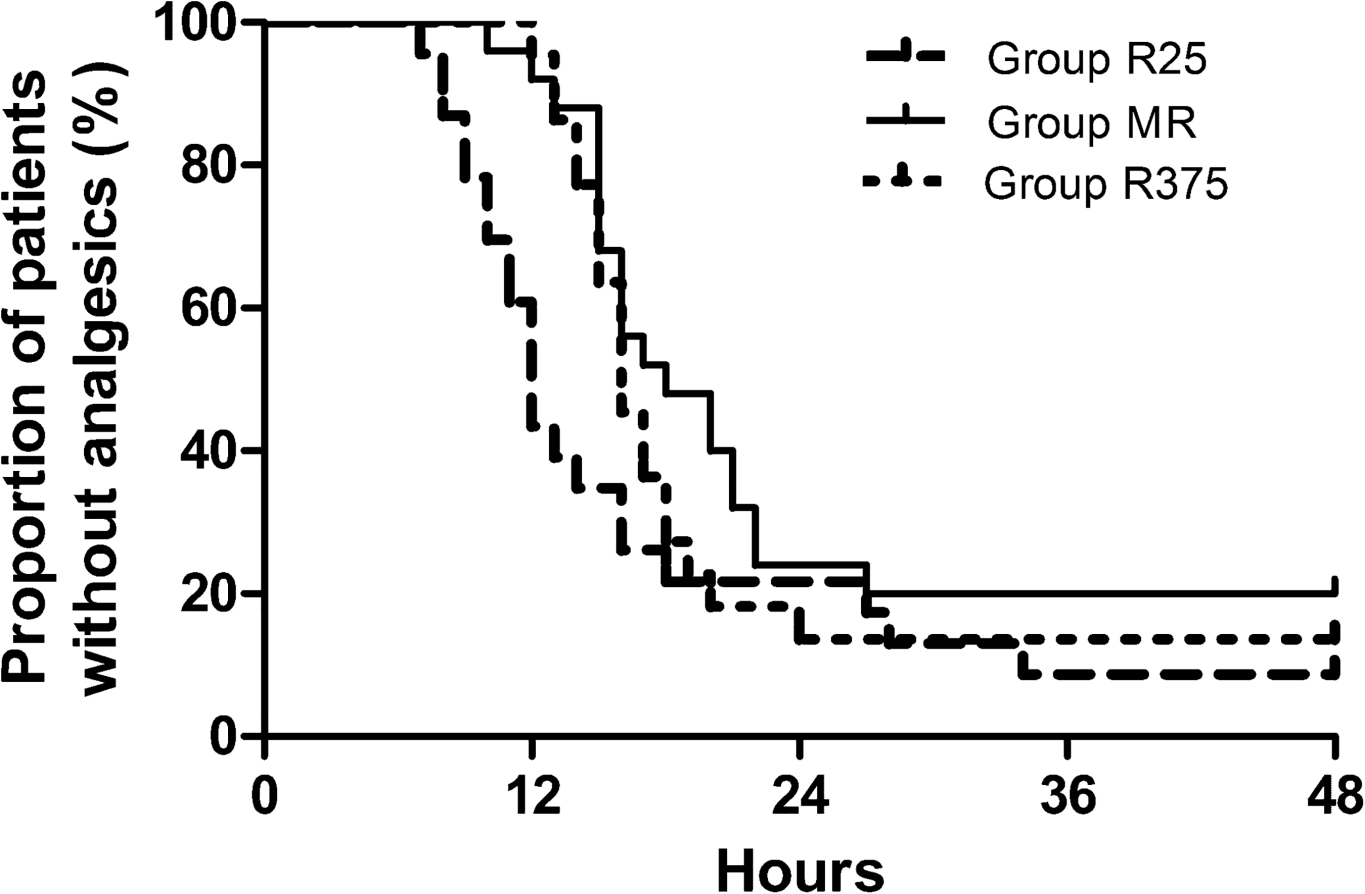
Kaplan-Meier curve analysis of postoperative analgesia: The initial time of analgesic requirement after operation. Group MR had a significantly longer time to first additional analgesic request compared with Group R25.

In comparison with Group R25, Group MR had no difference in the initial time of analgesic request after Bonferroni correction (P = 0.032) (Fig 3). Among the three groups, there were no significant differences in the number requiring rescue analgesics during the postoperative period. The total tramadol consumption over 24h was more in Group R25 in comparison with the other two groups(p = 0.001). The total tramadol consumption over 48h was less in Group MR in comparison with Group R25 (p = 0.001) and Group R375 (p = 0.002). The worst pain score in Group MR was less than that of Group R25 and Group R375 (p = 0.001). Patient satisfaction scores were greater in Group MR and Group R375 in comparison with Group R25 (P < 0.001). No significant difference was detected between Group MR and Group R375 for satisfaction score after Bonferroni correction (P = 0.028) (Table 3).

Reliable and satisfying anesthetic effect was achieved in all of the cases included in the analysis without additional fentanyl administration during the operation. Hemodynamic values did not differ among the three groups. Among the three groups, we did not observe any differences in the side effects such as shivering or nausea.

## Discussion

We showed that the addition of 200mg MgSO_4_ to 0.25% ropivacaine prolonged sensory block and improved evoked pain score compared with 0.25% ropivacaine alone. However, supplementary MgSO_4_ had no effect on the initial time of analgesic request and spontaneous pain intensity at rest. Interestingly, we found that the mean sensory block time for the R25 group was 8.73 hours (524.3min from Table 2). Nevertheless, patients in the group still felt evoked hyperalgesia when we probed the area close to clamed toes on 6^th^ hour after operation. It means that patients had hyperalgesia when ropivacaine still have worked on sensory block.

Hyperalgesia may cause worsening postoperative pain due to the amplification of noxious stimuli and sensitization. Several additives are being trialed in regional anesthesia practice with the goal of enhancing analgesic effect while minimizing unwanted effects (e.g., prolonged motor block). Clonidine added to local anesthetics has been demonstrated to prolong the duration of analgesia. However, meta-analysis showed that the increased risk of hypotension, fainting, and sedation limit its usefulness [10]. Dexamethasone could also prolong the duration of brachial plexus blocks [11]. However, hyperglycemia induced by dexamethasone limit its application in diabetic patient. Tramadol added to local anesthetics could prolong the duration of block while causing complications such as nausea, vomiting, seizure and increased sedation [12].

The specific involvement of NMDA receptors in peripheral blocks is less certain. Ketamine was reported to increase the frequency of adverse effects, such as hallucinations, drowsiness, and unpleasant feelings [13]. When added to local anesthetics for nerve block, MgSO_4_ could prolong duration of block [6], reduce postoperative analgesic requirements [6]. In chronic renal failure patients, the addition of MgSO_4_ to levobupivacaine shortened the time of onset of block, prolonged the total durations of sensory and motor block without exacerbating side effect over hemodynamic parameters [14]. In this trial, MgSO_4_ added to ropivacaine prolonged the duration of sensory block and reduced evoked pain intensity at the 6 hour observation in comparison with the same amount of ropivacaine (15ml of 0.25% ropivacaine) without supplementation of MgSO_4_.

Magnesium is believed to have an analgesic effect and opioid-sparing effect. However, systemic intravenous administration of magnesium did not increase cerebrospinal fluid (CSF) magnesium concentration and had no effects on postoperative pain [15]. How about potential neurotoxicity in magnesium for perineural injection? Actually, intravenous administration of magnesium has been reported to have neuroprotective effect in a variety of experimental models of brain [16] and spinal cord injury [17]. Large dose (3mg) of MgSO_4_ administered intrathecally caused significant neurodegeneration in rats [18]. However, low dose of magnesium (300ug) was safe in intrathecal administration [19]. Inadvertent administration of MgSO_4_ 9 g through an epidural catheter did not cause any signs of neurological toxicity in human [20]. Perineural application of MgSO_4_ may carry more risks. The dose of MgSO_4_ used in this study was proved safe before [6,8].

How MgSO_4_ enhance analgesic effect in a peripheral nerve block? The proposed mechanisms listed in the following. 1^st^, the enhanced analgesic effect of MgSO_4_ stems from the voltage-dependent antagonism of NMDA receptors, leading to the prevention of central sensitization from peripheral nociceptive stimulation [21]. 2^nd^, The increase in phasic block (with or without local anesthetics) in the presence of high Mg^2+^ concentration indicated that MgSO_4_ could slow the closing of Na^+^ channels and enhance the number of inactivated Na^+^ channels. Therefore, more local anesthetics molecule could reach the binding site on Na^+^ channels and block more Na^+^ channels [22]. 3^rd^, clinical investigations have shown that calcium channel blockers can potentiate the analgesic effect of local anesthetics [23]. Mg^2+^ was found to have calcium antagonism property that can regulate the calcium influx into the cell. 4^th^, hypomagnesemia is associated with CNS hyperexcitability as a result of increasing excitatory neuromediators and production of inflammatory mediators [22]. 5^th^, high concentration of Mg^2+^ in cellular membrane could cause hyperpolarization and nerve conduction block [22].

There was a strong correlation between hypomagnesemia and diabetic foot ulcers [24]. Due to vasoconstriction with inadequate blood supply [25] and loss of protective sensation [26], hypomagnesemia was found to associate with increased incidence of neuropathy and angiopathy in diabetic subjects [3,24].

The duration of sciatic nerve block was prolonged in type 2 diabetic patients with minor nerve injury. However, the prolonged block did not translate into an increased risk of nerve injury due to a limited study population [27]. Lowering the local anesthetic concentration in a diabetic patient is a worthy goal to improve safety.

The analgesic benefits of supplementary magnesium as shown in this study are modest. So should a combination of ropivacaine 0.25% and magnesium be chosen over ropivacaine 0.375%? In this study, the use of diluted local anesthetic with MgSO_4_ is not only equianalgesic with concentrated local anesthetic, but also is likely safer for patients. Some reviewers suggested using a lower dose of local anesthetic in the presence of severe diabetic peripheral neuropathy [28]. Local anesthetics may have toxic effect in diabetic nerves [29]. Actually nerve injected with 0.5% ropivacaine had a greater nerve fiber degeneration in diabetic rats than that of normal nerves. Adjuvant, like clonidine, could also aggravate the nerve degeneration in diabetic rats after nerve block [29]. In order to decrease local anesthetics induced neurotoxicity in diabetic patients, we decreased the concentration of ropivacaine to 0.375% and 0.25% respectively in this trial. The groups of 0.15% ropivacaine and 1% lidocaine were also employed in pre-test. However, patients in these groups were found to exhibit pain during operation, so we excluded these doses. This research focused on the analgesic adjuvant, in combination with less ropivacaine, to provide desired analgesia without peripheral nerve toxicity that diabetic patient have exhibited when exposed to traditional local anesthetics.

To avoid interference with inflammatory pain after operation, we examined the evoked hyperalgesia on the skin overlying the center of 3^rd^ metatarsus throughout this trial, which was at least 2 cm away from the edge of incision.

Limitations: In clinic, most of the diabetic patients who need amputation present with hypoalgesia, instead of hyperalgesia. Thus it was difficult to collect a sufficient number of patients for this trial. Some older patients who have cognitive dysfunction were excluded. This may create the age related bias for the outcome. Another limitation of this study is that it did not look for evidence of neurotoxicity in the study participants, which would require much more invasive follow-up and a much larger scale of study population. Due to lower dose of postoperative analgesics use and lower worst pain score in comparison with Group R375, Group MR may have higher satisfaction score in patients. However, this trial was under-powered to detect the statistical difference. However, we found that the satisfaction score in group MR was only 3.6 (on a scale that ranges from 0–100) higher than group R375. Besides, the difference of worst pain score between the group MR and group R375 was only 0.5 (on a 10-point scale). Such small differences were unlikely to be clinically meaningful. Anyway, group MR could achieve equianalgesic effect in comparison with group R375.

Group MR only decreased the value of NRS_evoked_ at 6 hour after operation and nearly all patients still needed additional rescue analgesics after operation. It was hypothesized that magnesium substitution was beneficial only in patients who had hypomagnesemia. In the current trial, there was no preoperative difference in serum magnesium level between each group. Future studies should focus on the diabetic patients with hypomagnesemia undergoing toe amputations. Although diabetic patients had no evidence of neurotoxicity in the trial, our study was not designed or powered to evaluate this outcome. More information about safety remains an issue for future studies.

In conclusion, perineural administration 0.25% ropivacaine with 200mg supplement of MgSO_4_ prolonged the sensory block when compared with 0.25% ropivacaine alone in diabetic patients. In comparison with Group R25, Group MR decreased the evoked pain score at 6 hour, reduced the dose of analgesic requirement postoperatively, and improved the worst pain score as well as satisfaction scores. In comparison with Group MR, the only advantage in Group R375 was less evoked pain score at 6 hour postoperatively. However, Group R375 even had higher score of worst pain score and larger dose of postoperative analgesics consumption. In the future research, we should compare the analgesic effect of ropivacaine + perineural Mg vs. ropivacaine + intravenous Mg. It might show a way to avoid potential (or unknown) neurotoxicity of perineural Mg while maintaining improved block quality.

## Supporting information

S1 File Trial protocol in English edition. (DOCX)

S2 File Trial protocol in Chinese edition. (DOCX)

S1 Table CONSORT 2010 checklist of the randomized trial. (DOC)
