# Supplementary material for: Analgesic effect of perineural magnesium sulphate for sciatic nerve block for diabetic toe amputation: A randomized trial
Source: PLoS One. 2017 May 2;12(5):e0176589. doi: 10.1371/journal.pone.0176589 (PMC5413065; doi:10.1371/journal.pone.0176589)
Supplement: S1 File — (DOCX) [file pone.0176589.s001.docx]

Clinical trial

Magnesium used for postoperative analgesia after diabetic toe amputation

1st affiliated hospital of Wenzhou Medical University

Jiehao Sun，Le Liu, Xiaona Feng，Qihan Zhu，Wendong Lin，Hailei Guo

Contacts：Le Liu, Jiehao Sun

1. background and purpose:

Incidence of DM is more and more now, accompanied with more DM induced complication. DM induced cardiovascular disease, embolism, pneumonitis can produce difficult in postoperative analgesia. DM induced hyperalgesia can cause psychological disorder and decrease satisfaction after operation.

Magnesium sulphate can inhibit NMDA receptor, inhibit adhesion of inflammatory cell, inhibit release of inflammatory factors, decrease central sensitization, and hence relive pain after surgery.

Patients in this trial is characterized by aged, complicated complication, which is difficult in analgesia. We will perform the sciatic nerve block guided by ultrasound B with the agent of ropivacaine and magnesium sulphate combination. We hope magnesium can prolong the duration of analgesia, decrease the dose of rescue analgesics after operation and increase patients’ satisfaction.

2.Design

This is a kind of prospective randomized, double blinded trial. 63 cases will be involved in the trial (21 cases per group, 3 groups in total). Randomization codes are generated by computer (Excel 2003).

3.Inclusion criteria

Diabetic foot patients who had hyperalgesia to stimulus indicating toe amputation were recruited into the clinical trial.

ASA: 1-2, age:50-70, operation time <1.5 hour, no gender limitation.

4.Exclusion criteria

The patients were excluded if they had no allodynia after stimulation. Other exclusion criteria included skin overlying the 3rd metatarsus was not intact; patient incapacitation to the trial; systemic disease that contraindicated participation in the study; sedative abuse; opioid abuse or alcohol abuse; allergic to test drugs; age >70yr or < 50 yr; hearing loss; unwilling to participate in the trial.

5.Drop out of cases

Drop out of cases include: failure to get data during the trial, quit the trial during operation, and any other situation who can not fulfill the trial.

If the patients is quit for the reason s like allergic reaction, side effect, ineffective analgesia, patients should be treated effectively.

6.When to terminate the trial

(1) severe side effect during the trial；

(2) severe error in the design of the trial.

(3) difficult to perform the trial in reality.

(4) poor clinical effect which can not be applied in clinics

(5) terminate the trial due to inadequate funding.

(6) terminate the trial by Department of Health Administration.

7. Trial protocol

7.1 Drugs

Magnesium sulphate 20ml （200mg）（Changzhou phar cor）

Ropivacaine injection 10ml (75mg) （astrazeneca）

7.2 Randomization

Randomized codes are generated from Microsoft Excel 2003. Each code will be sealed in a envelop which will be opened once the patient is enrolled. The envelops with codes were concealed at the Clinical Trial Center.

7.3.trial allocation

63 cases allocated into 3 groups:：

1) Group MR: n=21,perineural 200 mg MgSO4 added to 0.25% ropivacaine (15ml).

2) Group R25: n=21,0.25% ropivacaine alone (15ml).

3) Group R375: n=21,0.375% ropivacaine alone (15ml).

7.4 Procedure of the trial

Upon hospitalization, patients will be educated about Numeric Rating Scale of Pain intensity - Visually (NRS), for the purpose of becoming familiar with the method of pain degree evaluation. Routine monitoring of electrocardiogram, pulse oximetry, non-invasive blood pressure, heart rate is applied. Supplemental oxygen will be given by facemask (100% O2, 4 L/min) when required to maintain saturation above 92 % throughout the duration of the study.

An anesthesiologist will perform popliteal sciatic nerve block By using an ultrasound scanner with a 38 mm 6-13 MHz linear probe (EDGE, Fujifilm, SonoSite).The anesthesiologist is unaware of which regimen was administered Prior to surgery, no premedication was administered.

Sensory block is assessed for sciatic nerve cutaneous innervation area by pinprick test in 3 categories (0: normal sensation; 1: no pain but presence of tactile sense; and 2: no pain at all). Motor block is also assessed according to ankle dorsiflexion in 3 categories (0 = normal dorsiflexion strength; 1 = diminished dorsiflexion strength; 2 = totally absent strength.) The onset times of the sensory and motor blockades are defined as the time interval between the end of local anesthetic administration and the loss of sensation to pinprick (sensory score = 1) and absent movement (motor score = 2), respectively.

7.5.side effect during operation

(1) Hypotension was defined as systolic blood pressure < 90 mm Hg or > 30% decrease from baseline. 30mg of ephedrine should be given, following by more fluid infusion.

(2) Hypertension was defined as systolic blood pressure ＞180 mmHg or > 30% increase from baseline. 0.25～0.5mg nitroglycerin should be given, following by less fluid infusion.

(3) Bradycardia was defined as < 50 beats/min. 0.2～0.5 mg atropine should be given.

(4) Tachycardia was defined as ＞110 beats/min. 0.5 mg esmolol should be given.

(5) Intraoperative pain with NRS > 3 would be treated with increment dose of intravenous fentanyl 50ug.

8. Outcome measures

8.1 Baseline

(1) gender, age, height, weight、duration of DM、duration of diabetic foot.

(2) History of Present Illness, concurrent disease, allergic reaction, hypertension history.

(3) vital sign before anesthesia: RR, SBP,DBP,MAP,HR,SpO2.

8.2 Experimental drug efficacy

8.2.1postoperative analgesia: NRS score、initial time of rescue analgesics、consumption of rescue analgesics(tramadol)、time of rescue analgesics、patients’ satisfaction score.

8.2.2 NRS score：make a record of NRS score after operation (0、6、24、48h). make a record of the worst pain score after operation.

NRS score：0: no pain，10:untolerable pain（1～3:mild pain；4～6:moderate pain；7～10:severe pain）.

The primary outcome was the NRS score after the operation, time to first request of rescue analgesics.

Secondary outcomes included duration of block, patient satisfaction and the worst pain score.

8.3 side effect

8.3.1 Hemodynamic changes：make a record of vasoactive drugs during the operation.

8.3.2 Respiration：Supplemental oxygen was given by facemask (100% O2, 4 L/min) when required to maintain saturation above 92 % throughout the duration of the study.

8.3.3 Nausea：make a record of cases who have nausea during the operation.

8.3.4 Others: Dry mouth, shivering, delirium, itching, et al.

9.Complication

9.1 Common complication induced by magnesium sulphate.

9.1.1 Hypotension: systolic blood pressure < 90 mm Hg or > 30% decrease from baseline.

9.1.2 Bradycardia: Heart beat < 50 beats/min；

9.1.3 Respiratory depression: respiratory frequency ＜8 bpm; or duration of respiratory standstill > 15 seconds.

9.1.4 Nerve injury: sensory and motor have not reversed 24 hour later. EMG should ba applied to confirm the nerve injury.

9.2 How to confirm the drug induced adverse reaction

(1) The time of adverse reaction is in accordance with the time of administration.；

(2) Adverse reaction is associated with the known side effect.

(3) Adverse reactions can not be explained by other reasons;

(4) Adverse reaction could be alleviated when the drug is withdrawn.

(5) Adverse reaction will come back when the drug is given again.

10.The duration of the trial：

From March 2014 to August 2015

11.Expected benefit from the trial

Cases allocated to one group will benefit in less pain and uncomforting after operation.

12.Risks involved in the trial

We expect no risks involved in the trial. Perineural administration of magnesium was given before, and no toxicity was observed in the trials. Actually, magnesium have neural protection in neural and spinal injury animals.

Any discomfort will be treated during the trial.

13.Drugs involved the trial and the charge for the drugs

The drugs involved in the trial was permitted by Chinese drugs agency. Magnesium sulphate(Changzhou Pharma Cor) was free for use in the trial, as well as the side effect induced by the drug. We do not provide any other financial support for the patients in the trial.

63 cases

NRS education

Patients’ satisfaction

Worst pain score after operation

Group R375: 21

Group R25: 21

Group MR: 21

Popliteal sciatic nerve block guided by ultrasound B(15 ml)

Postoperative complication

NRS score after operation (0,6,24,48 hour)

Initial time of rescue analgesics, dose of rescue analgesics,
