# Supplementary material for: Analgesic effect of perineural magnesium sulphate for sciatic nerve block for diabetic toe amputation: A randomized trial
Source: PLoS One. 2017 May 2;12(5):e0176589. doi: 10.1371/journal.pone.0176589 (PMC5413065; doi:10.1371/journal.pone.0176589)
Supplement: S2 File — (DOCX) [file pone.0176589.s002.docx]

临床试验标书

硫酸镁用于糖尿病足术后镇痛

温州医科大学附属第一医院

孙捷豪，刘乐，冯晓娜，朱启涵，林文东，郭海雷

负责人：刘乐，孙捷豪

1. 研究背景和研究目的

现在随着人民生活水平的提高,糖尿病的发病率也大大提高,糖尿病并发症如糖尿病足患者大大增多。糖尿病患者身体合并症众多，像心脑血管疾病，血管栓赛等，这些疾病可导致患者肺炎，心脑血管意外，肝素化导致的血肿等术后并发症增多，部分糖尿病患者存在痛觉超敏，使术后镇痛变得棘手，甚至导致住院时间延长、住院费用增加，严重影响患者的生活质量。

硫酸镁注射液可通过抑制抑制NMDA受体,抑制炎性细胞与血管内皮细胞黏附从而减轻术后的注射痛。通过研究硫酸镁能抑制NMDA受体，减少局部组织的炎症反应，减少炎症因子释放，减少痛觉的中枢型敏化。本研究观察的糖尿病足患者，年龄较大，合并症多，术后疼痛处理棘手，不适合全身麻醉，我们将硫酸镁复合罗哌卡因腘窝处坐骨神经阻滞，以减少术后疼痛,减少炎症刺激因子的释放,首次给予补救性镇痛药物的时间延长，围术期镇痛药物使用量减少。

2.试验设计

本实验采用前瞻性、随机、盲法、安慰剂对照临床研究方法。本试验的试验药物均为注射剂，共观察63例病例（每组21例，共3组）。由计算机产生随机号。

3.哪些人适合参加研究

痛觉超敏的糖尿病足患者，行糖尿病足截肢术，ASA 1-2级，年龄50-70岁，手术时间< 1.5小时，性别不限。

4.哪些人不宜参加研究

以下患者不适于参加本研究：听觉、视觉障碍；精神疾病史；长期服用镇静剂；嗜酒、吸毒史；预期生存率小于12个月；心力衰竭，EF小于等于30%，或6个月内因心力衰竭入院者；12个月内有中风史；既往12个月内有严重的出血史；对硫酸镁或利多卡因过敏；30天内正在参加其他临床试验的患者；年龄>70yr 或< 50 yr;。另外还有1）正参加其它临床试验的患者；2）研究人员认为其他原因不适合临床试验者。

5.脱落标准

无论何时何因退出，只要没有完成方案所规定的观察周期，均称为脱落病例；

因过敏反应、不良反应、治疗无效而退出时，研究者应根据受试者实际情况，采取相应的治疗措施。

6.终止试验的条件

(1) 试验中发生严重不良事件，应及时中止试验；

(2) 试验中发现临床试验方案有重大失误;

(3) 方案在实施中发生严重偏差，难以评价药物疗效，应中止试验；

(5) 试验中发现药物治疗效果（实验药物的镇痛、镇静作用）较差（无治疗作用或与对照组相比，各观察指标差异无统计学意义），不具备临床价值，应中止试验；

(5) 申办者因资金等原因要求中止试验；

(6) 卫生行政主管部门撤销试验。

7、实验方案

7.1 试验药物

试验药：硫酸镁 20ml （200mg）（常州药物有限公司）

罗哌卡因 10ml (75mg) （阿斯利康有限公司）

7.2随机化分组

本实验使用excel随机产生随机号，并由本临床实验中心不参与给药和疗效观察的人员密封在信封中，由实验中心统一保管。

7.3 病人分组

将63例患者随机分成3组：

硫酸镁罗哌卡因组（MR组，n=21），即200mg硫酸镁与0.25%罗哌卡因注射液15ml。

罗哌卡因25组（R25组，n=21），即0.25%罗哌卡因注射液15ml。

罗哌卡因375组(R375组,n=21)，即0.375%罗哌卡因注射液15ml。

7.4 实验步骤

患者住院期间，宣教使之熟悉NRS的评分，两组患者均于术前禁食，进入手术室后常规开放外周静脉，监测心电图（ECG）、无创血压（NBP）、心率（HR）、血氧饱和度（SPO2），当血氧饱和度下降至92%以下时，面罩吸氧(100% O2, 4 L/min)。

麻醉医生用超声38 mm 6-13 MHz (EDGE, Fujifilm, SonoSite)引导进行腘窝坐骨神经阻滞，该麻醉医生不清楚具体哪种药物进行注射。术前不给予任何静脉辅助用药。

坐骨神经感觉阻滞的测定分3级：（1：正常感觉2：有触压觉，无痛觉3：没有任何感觉）。坐骨神经运动阻滞的测定根据踝背屈分3级：（1：正常背屈2：背屈减弱3：不能背屈）。药物阻滞感觉和运动的起效时间是从给予药物开始算，一直到感觉阻滞(感觉评级 = 1)和运动阻滞(运动评级 = 2)起效。

7.5 手术期间并发症

(1) 如果SBP＜80 mmHg，或下降幅度超过基础血压的30%，静脉给予麻黄碱30 mg，并加快液体的输注速度，必要时重复；

(2) 如果SBP＞180 mmHg，减慢输液，静注硝酸甘油0.25～0.5mg或尼卡地平0.1～0.2 mg，必要时重复；

(3) 如果心率＜50次/分，静注阿托品0.2～0.5 mg，必要时重复；

(4) 如果心率＞110次/分，静注艾司洛尔0.5mg/kg，必要时重复；

(5) 如果镇痛不全，肌注芬太尼50 ug/次。

8、观察指标

8.1 一般资料

(1) 记录患者：性别、年龄、身高、体重、糖尿病时间、糖尿病足时间。

(2) 记录患者：病史、现病史、合并疾病及用药、过敏史、高血压病史（最高血压、服药种类及年限、有无其他脏器合并症）。

(3) 记录患者：入室时生命体征：呼吸次数、无创血压（SBP、DBP、MAP）、HR、SpO2。

8.2 试验药物有效性指标

8.2.1术后镇痛情况，NRS评分、首次补救药物镇痛时间、补救药物的使用次数，补救药物（曲马多）总剂量，患者的满意度作为药物有效性的衡量指标，

8.2.2 疼痛评分及镇静评分：记录手术后0、6、24、48h时疼痛评分（NRS）。记录最痛时刻的NRS评分

NRS评分：0 分无痛，10 分为无法忍受的剧痛（1～3分为轻度疼痛；4～6分为中度疼痛；7～10分为重度疼痛，病人往往无法入睡）。

主要有效性指标: NRS评分、首次补救药物镇痛时间

次要有效性指标：镇痛药（舒芬太尼）消耗量、补救药物的使用次数，补救药物总剂量，患者的满意度作为药物有效性的衡量指标，

8.3 安全性指标

8.3.1 血流动力学改变：记录给予血管活性药物的次数和剂量。

8.3.2 呼吸监测：吸空气SpO2<92%时呼唤患者并面罩吸氧（4L/min）。

8.3.3 恶心：记录发生恶心的病例人数。

8.3.4 其他不良事件：如口干、寒颤、谵妄、皮肤瘙痒等。

9.常见不良反应及不良事件的界定

9.1 试验药物硫酸镁的常见不良反应

(1) 低血压、心动过缓及窦性停搏；收缩压＜90 mmHg或比试验药物输注前值低30%以下，或舒张压＜50 mmHg；

(2)心动过缓的定义为：心率＜50 bpm；

(3) 呼吸抑制的定义为：呼吸频率＜8次/min或呼吸暂停时间>15s；

(4) 神经损伤：24小时后神经感觉或运动神经未恢复，需要做肌电图确认

9.2 不良反应评价标准

(1) 不良反应出现的时间与用药时间吻合；

(2) 不良反应与该药已知不良反应有关

(3) 不良反应不能用其他原因解释；

(4) 不良反应停药后减轻或消失；

(5) 不良反应在给药后再现。

10．研究起始时间：

2014年03月到2015年08月。

11.参加研究可能的受益

参加本研究的受试者，您被分配到的某个组别可能因为合适的镇痛方案减少您的术后疼痛。

12.参加研究可能的不良反应、风险和不适、不方便

我们的研究设计是期望能够减少糖尿病足的术后疼痛及其并发症。但是由于随机分组的问题，我们不知道患者是否能够进入术后疼痛最低的那一组，但是我们的设计不会在原来的基础上增加您的不良事件率。

我们希望我们的试验不会引起任何副作用。硫酸镁用于神经阻滞已经被多个试验报道，目前没有相关毒性反应，实际上，硫酸镁甚至还可以对脑和脊髓损伤的动物模型中有保护作用。

如果在研究期间您出现任何不适，或病情发生新的变化，或任何意外情况，不管是否与研究有关，均应及时通知您的医生，他/她将对此作出判断并给与适当的医疗处理。

13.有关费用

参加本研究的受试者，将获得减少手术术后疼痛的益处。因为我们所应用的研究药物是上市后的药物，且它的使用剂量及使用方法符合药物的使用说明。该药物的安全性是得到中国权威部门认可的。故与研究药物有关的住院及治疗费用我们将不提供，由您的医疗保险承担。我们研究应用的研究药物是常州医药有限公司生产的药物，她的产品本身就是符合国家标准并且是上市销售的。

技术路线：

63例患者

术前进行NRS评分教育

术后0，6，24，48小时记录疼痛NRS评分

首次用镇痛药物的时间及镇痛药物总量

腘窝处坐骨神经注射相应药物15ml

R375组21例

R25组21例

MR组21例

术后48小时评价患者满意度满意度，患者最痛时刻的NRS评分

并发症出现情况
